# Generation and Characterization of Induced Pluripotent Stem Cells and Retinal Organoids From a Leber’s Congenital Amaurosis Patient With Novel *RPE65* Mutations

**DOI:** 10.3389/fnmol.2019.00212

**Published:** 2019-09-11

**Authors:** Guilan Li, Guanjie Gao, Panfeng Wang, Xiaojing Song, Ping Xu, Bingbing Xie, Tiancheng Zhou, Guangjin Pan, Fuhua Peng, Qingjiong Zhang, Jian Ge, Xiufeng Zhong

**Affiliations:** ^1^State Key Laboratory of Ophthalmology, Zhongshan Ophthalmic Center, Sun Yat-sen University, Guangzhou, China; ^2^Key Laboratory of Regenerative Biology, South China Institute for Stem Cell Biology and Regenerative Medicine, Guangzhou Institutes of Biomedicine and Health, Chinese Academy of Sciences, Guangzhou, China; ^3^Department of Neurology, Third Affiliated Hospital of Sun Yat-sen University, Guangzhou, China

**Keywords:** reprogramming, differentiation, retinal organoids, *RPE65* gene mutations, retinal degeneration

## Abstract

RPE65-associated Leber congenital amaurosis (LCA) is one of highly heterogeneous, early onset, severe retinal dystrophies with at least 130 gene mutation sites identified. Their pathogenicity has not been directly clarified due to lack of diseased cells. Here, we generated human-induced pluripotent stem cells (hiPSCs) from one putative LCA patient carrying two novel *RPE65* mutations with c.200T>G (p.L67R) and c.430T>C (p.Y144H), named RPE65-hiPSCs, which were confirmed to contain the same mutations. The RPE65-hiPSCs presented typical morphological features with normal karyotype, expressed pluripotency markers, and developed teratoma in NOD-SCID mice. Moreover, the patient hiPSCs were able to differentiate toward retinal lineage fate and self-form retinal organoids with layered neural retina. All major retinal cell types including photoreceptor and retinal pigment epithelium (RPE) cells were also acquired overtime. Compared to healthy control, RPE cells from patient iPSCs had lower expression of RPE65, but similar phagocytic activity and VEGF secretion level. This study provided the valuable patient specific, disease targeted retinal organoids containing photoreceptor and RPE cells, which would facilitate the study of personalized pathogenic mechanisms of disease, drug screening, and cell replacement therapy.

## Introduction

Leber’s congenital amaurosis (LCA) is a group of recessively inherited retinal dystrophies (IRDs) with severe visual impairment, accounts for >5% of all retinal dystrophy and 20% of legal blindness in school-age children. The disease is characterized by vision loss from birth or the first few months of life verified by electroretinogram (ERG) recording with markedly reduced or undetectable rod and cone response, nystagmus, poor pupil light reflex, and variable fundus changes from normal retinal appearance to severe pigmentary degeneration. The estimated prevalence is 2–3 per 100,000 people worldwide (Koenekoop, 2004; Coussa et al., 2017). To date, at least 20 mutation genes such as *CEP290*, *GUCY2D*, *CRB1*, and *RPE65* have been identified in patients with LCA. These genes were proved to express in photoreceptors or retinal pigment epithelium (RPE) cells of retina, and involved in disparate functional pathways including photoreceptor morphogenesis, visual phototransduction, and visual cycle (Wang et al., 2015; Jacobson et al., 2016; Kumaran et al., 2017).

RPE65 is a 65 kDa isomer hydrolase synthesized in RPE cells. It catalyzes isomerization of all-trans retinyl esters into the chromophore 11-*cis* retinol which is transported into photoreceptors to participate in visual phototransduction (Jin et al., 2005). This process is also called retinoid cycle or visual cycle. Mutations in this gene not only disrupt this functional visual cycle, but also lead to structural degeneration of outer neural retina (NR) and RPE over time, causing irreversible blindness, including LCA, retinal pigmentosa (RP), or cone-rod dystrophies (CORDs) (Bereta et al., 2008). Comprehensive genotyping identified *RPE65* as one of the most prevalent mutated genes in LCA patients, accounting for approximately 3–16% of all LCA cases with the highest in the Caucasian and India population. Although many variants in this gene have been documented, their pathogenicity have not been directly clarified partly due to lack of human *in vitro* disease models (Astuti et al., 2016; Bernardis et al., 2016).

So far, there is no cure for IRDs including LCA. With the advancement of gene and stem cell technology, both gene and cell therapy have been regarded as emerging, promising therapeutics for this kind of diseases (Wiley et al., 2015; Dalkara et al., 2016). RPE65 gene therapy completed phase 3 clinical trial by Russell et al. (2017), and got approved firstly by FDA for the treatment of RPE65-mediated IRD in the United States (Bainbridge et al., 2008). Although treatment effects have been observed with improved light sensitivity and mobility after subretinal administration of AAV-*RPE65* gene complex in both animal models with RPE65 mutations and RPE65-LCA patients, the improved visual function started declining 3 years after treatment and this gene therapy approach could not prevent progress of retinal degeneration with photoreceptor apoptosis, which eventually leads to retinal cell loss including RPE cells and photoreceptors. In addition, having sufficient viable retinal cells has become a prerequisite for successful gene therapy in patients with RPE65 mutations and in other retinal degenerative conditions (Dalkara et al., 2016). Therefore, cell therapy would be in demand alone or combined with gene therapy for replacing the lost or diseased retinal cells to recover the visual function and retinal structures simultaneously, especially in advanced retinal degenerative conditions.

Ten years ago, human induced pluripotent stem cells (hiPSCs) were reprogrammed from human somatic cells with Yamanaka’s four transcription factors (Takahashi et al., 2007). This technology provides opportunities for study and treatment of degenerative diseases in a subject-personalized manner since hiPSCs have capacity to differentiate into almost all body cells including retinal cells (Meyer et al., 2009; Zhong et al., 2014). More importantly, human iPSCs can be directed step by step into three-dimensional (3D), laminated retinal organoids containing all major retinal cells located in proper layers with photoreceptors achieving quite high degree of maturation, resembling human retinal development *in vivo* (Reichman et al., 2014; Zhong et al., 2014; Li et al., 2018). The iPSC-3D retinal organoid induction approach provides not only unlimited cell source for retinal cell replacement therapy, but also a powerful platform for disease modeling, drug screening, and even preclinical testing of gene therapy for IRD.

In this study, we established LCA patient-specific iPSC lines with two mutations c.200T>G (p.L67R) and c.430T>C (p.Y144H) in *RPE65*, reprogrammed from urine epithelium cells. Under retinal differentiation conditions, the patient-specific iPSCs were able to differentiate into retinal organoids with laminated NR and RPE cells. Their cellular and molecular features were similar to those differentiated from control hiPSCs. However, compared with healthy control, the RPE65 expression level was decreased in patient RPE cells while phagocytosis and VEGF secretion activity were equivalent. The patient-specific retinal tissues might serve as a valuable source or disease model for personalized study and treatment.

## Materials and Methods

### Case Patient

A LCA patient was diagnosed by clinical standards with compound heterozygotes *RPE65* gene mutations (c. [200T>G], p. L67R; c. [430T>C], p. Y144H) as reported by Chen et al. (2013). This study was approved by the ethics committee of the Zhongshan Ophthalmic Center of Sun Yat-sen University and was conducted in accordance with the Declaration of Helsinki. The patient agreed to take part in this experiment and signed informed consent. The clinical features and genotype of this patient have been identified before (Chen et al., 2013).

### Urine Collection and Cell Expansion From a Patient With RPE65-LCA

Collection and expansion of urine cells (UCs) was performed as described previously (Zhou et al., 2011). Briefly, the mid-stream urine (100–200 ml) was collected into sterile containers from the RPE65-LCA patient. The urine samples were centrifuged at 400 × *g* for 10 min. Cell pellet was washed with PBS containing amphotericin B and 100 U/ml penicillin/streptomycin and resuspended in 2 ml of primary medium consisting of DMEM/Ham’s F-12 nutrient mix (1:1) (Thermo Fisher Scientific, Waltham, MA, United States), 10% of fetal bovine serum (FBS) (Natocor, Villa Carlos Paz, Cordoba, Argentina), renal epithelial cell growth medium (REGM) SingleQuot kit supplement (Lonza, Basel, Switzerland), amphotericin B, and 100 U/ml of penicillin/streptomycin. The cells were seeded into a 12-well plate coated with 0.1% gelatin and switched to REGM (Bullet Kit, Lonza, Basel, Switzerland) 4 days later. Adherent cells/colonies appeared after 3–6 days, passaged by TrypLE express (Life Technologies, Inc., Grand Island, NY, United States) when cell density reached 80–90% confluence.

### Urine Cell Reprogramming and hiPSCs Culture

The method used to reprogram UCs into human iPSCs was described previously (Xue et al., 2013) with slightly modifications. In short, 1 × 10^6^ UCs of passage 2 were electroporated with 6 μg OriP/EBNA1-based episomal plasmid pEP4EO2SET2K (contains OCT4, SOX2, SV40LT, and KLF4) and 4 μg pCEP4-miR-302-367 cluster (contains miR-302b, c, a, d, and miR-367) by Electroporation System (Lonza, Program T-020, Basel, Switzerland). Transfected cells were plated onto Matrigel-coated six-well plates and cultured in REGM. During D2–D16, induced medium mTeSR1 (Stem Cell Technologies, Vancouver, BC, Canada) containing 0.5 μM A-83-01 (SML0788, Sigma), 3 μM CHIR99021 (S1263, Selleck), 0.5 μM Tzv (S1459, Selleck), and 0.5 μM PD0325901 (S1036, Selleck) was changed every other day. The identifiable hiPSCs colonies with clear boundary were manually picked up during 16–21 days, and cultured in mTeSR1 on Matrigel-coated surface. Cells were passaged at ∼80% confluence with 0.5 mM EDTA (Invitrogen) from passage 2. Healthy control hiPSCs lines UE022 and UE017 were gifts from Professor GP (Guangzhou Institutes of Biomedicine and Health, Chinese Academy of Sciences).

### Retinal Differentiation and 3D Retinal Organoids Culture

Retinal differentiation with RPE65-hiPSCs was performed with published protocols with a slight modification (Zhong et al., 2014; Li et al., 2018). Briefly, on Day 0 (D0), hiPSCs were digested into small clumps and cultured in suspension with mTeSR1 and 10 μM Blebbistatin (Sigma-Aldrich) to form embryoid bodies (EBs). Neural induction medium (NIM) containing DMEM/F12 (1:1), 1% N2 supplement (Invitrogen), 1% non-essential amino acids (NEAA), 2 μg/ml heparin (Sigma-Aldrich) was used and changed with a 3:1 ratio of mTeSR1/NIM on D1, 1:1 on D2, and 100% NIM on D3. EBs were plated on Matrigel-coated dishes containing NIM on D5–D7. On D16, the culture medium was changed to retinal differentiation medium (RDM) [DMEM/F12 (3:1) supplemented with 2% B27 (without vitamin A, Invitrogen), 1% NEAA, and 1% antibiotic–antimycotic]. In 4–6 weeks after differentiation, horseshoe-shaped NR domains along with the surrounding RPE cells were manually detached with a sharpened Tungsten needle and subject to suspension culture for the formation of retinal organoids. For long-term culture, the organoids were switched to retinal culture medium (RCM) containing RDM, 10% FBS, 100 μM Taurine (Sigma-Aldrich), and 2 mM GlutaMAX in 1 week after detachment. Since Week (W) 13 and onward, B27 in RCM was replaced with N2. Medium was changed every 2–3 days.

### Isolation and Culture of RPE Cells

After NR domains were picked out, most RPE cells were left behind and kept growing for about 1 month, and then detached from the adherent surface, digested into single cells with TrypLE Express (Life Technologies, CA, United States) for 5–10 min in a 37°C incubator. The individualized RPE cells were plated on Matrigel-coated plates containing RCM, and passaged once reaching ∼90% confluence. To promote maturation, the confluent RPE cells on D7 after passage were switched to RDM again. The control RPE derived from healthy hiPSCs line UE022 were cultured in the similar manner as described above.

### Immunocytochemistry

Cells growing on coverslips were fixed in 4% paraformaldehyde (PFA, Sigma-Aldrich) for 5–10 min. Retinal organoids were fixed in 4% PFA for 30 min and dehydrated in gradient sucrose solutions 6%, 12.5%, 25% in turn. Tissue embedding, sectioning, and immunohistochemistry were performed as previously described (Zhong et al., 2014; Li et al., 2018). Briefly, cells or sections were permeabilized and blocked with 0.25% Triton X-100 and 10% donkey serum for 1 h at room temperature, then incubated with primary antibodies at 4°C overnight and incubated with the corresponding secondary antibodies with either Alexa Fluor 488 or 555 (Life Technologies, CA, United States) for 1 h at room temperature. DAPI (Sigma-Aldrich) was used to counterstain nuclei. Sections stained with the corresponding secondary antibody alone were used as negative controls. Fluorescence images were acquired with an LSM 510 confocal microscope (Zeiss, Jena, Germany). Both primary and secondary antibodies used are listed in Table 1.

**TABLE 1:** List of antibodies used for immunofluorescence staining.

**Antibody names**	**Company**	**Catalog number**	**Work concentration**
**First antibody**			
E-cadherin	Abcam	ab76055	1:500
KRT7	GeneTex	GTX107343	1:500
CD44	GeneTex	GTX102111	1:500
OCT4	Bioss	bs-0830R	1:250
SOX2	Abcam	ab92494	1:100
NANOG	Abcam	ab24624	1:1000
SSEA4	Abcam	ab16287	1:200
TRA-1-81	Abcam	ab16298	1:100
TRA-1-60	Abcam	ab16288	1:100
PAX6	DSHB	3B5	1:50
SOX1	Abcam	ab109290	1:200
SIX3	Rockland	600-401-A26	1:500
OTX2	Abcam	ab21990	1:500
LHX2	Santa Cruz	sc-81311	1:200
VSX2	Millipore	ab9016	1:500
MCM2	Abcam	ab4461	1:1000
BRN3	Santa Cruz	SC-6026X	1:200
AP2	DSHB	3B5a	1:35
PROX1	Millipore	AB5475	1:2000
PKC-α	Abcam	ab32376	1:2000
CRALBP	Abcam	ab15051	1:200
Recoverin	Millipore	ab5585	1:500
Rhodopsin	Abcam	ab3267	1:200
L/M opsin	Gift from Dr Jeremy	Nathans	1:5000
S opsin	Gift from Dr Jeremy	Nathans	1:5000
ZO-1	Thermo Fisher	33-9100	1:400
RPE65	Abcam	ab78036	1:400
MITF	Abcam	ab12039	1:400
**Second antibody**			
donkey anti-rabbit (555)	Invitrogen	A31572	1:500
donkey anti-rabbit (488)	Invitrogen	A21206	1:500
donkey anti-mouse (488)	Invitrogen	A21202	1:500
donkey anti-mouse (555)	Invitrogen	A31570	1:500
donkey anti-goat (555)	Invitrogen	A21432	1:500
donkey anti-sheep (488)	Invitrogen	A11015	1:500

### Reverse Transcription – PCR and qRT-PCR

Total RNA was extracted using Trizol (Invitrogen), and complementary DNA (cDNA) was synthesized from 500 ng of total mRNA using Prime Script^TM^ RT Master Mix (Takara Bio, Tokyo, Japan). PCR cycle program was: 95°C for 2 min, 35 cycles of 95°C for 30 s, 60°C for 30 s, and 72°C for 30 s, and final step was 72°C for 8 min. Subsequent PCR products were run on 2% agarose gels for 30 min. RT-PCR was performed with the primers listed in Table 2.

**TABLE 2:** Primer list.

**Genes**	**Size(bp)**	**Forward**	**Reverse**
**Exogenous genes (RPE65-hiPSCs) for RT-PCR**			
OCT4	657	AGTGAGAGGCAACCTGGAGA	AGGAACTGCTTCCTTCACGA
SOX2	534	ACCAGCTCGCAGACCTACAT	CCCCCTGAACCTGAAACATA
KLF4	401	CCCACACAGGTGAGAAACCT	CCCCCTGAACCTGAAACATA
SV40LT	491	TGGGGAGAAGAACATGGAAG	AGGAACTGCTTCCTTCACGA
ORIP	544	TTCCACGAGGGTAGTGAACC	TCGGGGGTGTTAGAGACAAC
EBNA-1	666	ATCGTCAAAGCTGCACACAG	CCCAGGAGTCCCAGTAGTCA
miR-302-367	322	TTTCCAAAATGTCGTAATAACCCCG	CTCCCAAAGAGTCCTGTTCTGTCCT
GAPDH	542	ACCACAGTCCATGCCATCAC	TCCACCACCCTGTTGCTGTA
**Enogenous genes (RPE65-hiPSCs) for RT-PCR**			
OCT4	323	CGAGCAATTTGCCAAGCTCCTGAA	TCGGGCACTGCAGGAACAAATTC
SOX2	448	CCCCCGGCGGCAATAGCA	TCGGCGCCGGGGAGATACAT
NANOG	237	AAGGTCCCGGTCAAGAAACAG	CTTCTGCGTCACACCATTGC
**Genes (retinal differentiation and RPE cells) for Q-PCR**			
GAPDH	120	TCGTGGAAGGACTCATGACC	AGGCAGGGATGATGTTCTGG
PAX6	120	AGT GAA TCA GCT CGG TGG TGT CTT	TGC AGA ATT CGG GAA ATG TCG CAC
VSX2	122	GGCGACACAGGACAATCTTTA	TTCCGGCAGCTCCGTTTTC
MITF	201	TGACCGCATTAAAGAACTAGGT	AGTTCCTGTATTCTGAGCAACA
RX	81	AGCGAAACTGTCAGAGGAGGAACA	TCATGCAGCTGGTACGTGGTGAAA
RPE65	259	GCCCTCCTGCACAAGTTTGACTTT	AGTTGGTCTCTGTGCAAGCGTAGT
CRALBP	173	GCTGCTGGAGAATGAGGAAACTC	GGCTGGTGGATGAAGTGGAT

Quantitative real-time PCR (qRT-PCR) was performed using AB Applied Biosystems (step one plus). Reactions were performed in triplicate, and Ct values were calculated using the 2^–Δ^ ^Δ^ ^Ct^ method. D5 EBs were used as reference. The expression levels of target genes were normalized to that of internal control gene GAPDH. Primer sequences are listed in Table 2.

### Alkaline Phosphatase Staining

RPE65-hiPSCs were stained according to the AKP staining kit instructions (D001-2, Nanjing Jiancheng Bioengineering Institute, China).

### Karyotype Analysis

G-band staining of chromosomes was used for karyotype analysis. RPE65-hiPSCs were grown on Matrigel-coated six-well plates until reaching 70% confluence. Colchicine was added to a final concentration of 0.2 μg/ml for 2 h. Then, RPE65-hiPSCs were digested with 0.5 mM EDTA solution for 5 min, collected and centrifuged at 1000 rpm for 5 min. Cell pellets were resuspended in 8 ml of 0.075 M KCl solution, incubated for 20 min at 37°C, and then fixed with 3:1 mixture of methanol/acid acetic solution for 10 min at 37°C. After further centrifugation, the supernatant was removed, and 10 ml ice-cold fixative solution was added. Cells were dropped on a cold slide, incubated at 80°C for 2 h, trypsinized, and then stained with Giemsa. Metaphase status was observed under an Olympus BX51 Microscope and analyzed with Ikaros Karyotyping System (MetaSystems).

### Sanger Sequencing

*RPE65* gene mutations in hiPSCs from the patient were verified by Sanger sequencing. The genomic DNA was extracted using a DNA Midi Kit (Qiagen, Valencia, CA, United States) according to the manufacturer‘s protocol.

### Modeling of RPE65 Structure With Mutations

The sequence of human RPE65 was obtained from UniProt^1^. The Crystal structure of bos taurus RPE65 (Protein Data Bank ID: 4RYZ) that displays 62% sequence similarity with the human protein was chosen as a template. The 3D homology model of human RPE65 was constructed using Swiss-Model (Biasini et al., 2014), and then optimized and mutated with FoldX 5.0 (Schymkowitz et al., 2005). Hydrogen bond and electrostatic potential analysis were done using VMD 1.9.3 (Humphrey et al., 1996) and PDB2PQR 2.1.1 (Dolinsky et al., 2004), respectively.

### Teratoma Formation Assay

For teratoma formation, 1–2 × 10^6^ hiPSCs with 30% Matrigel were injected intramuscularly into the hind limb of 6-week-old immunocompromised NOD-SCID mice. Animals were monitored each week and teratomas were dissected at W8–W10 after transplantation. The teratoma tissues were fixed in formalin, embedded in paraffin, and sectioned and stained with Hematoxylin and Eosin (HE). Images were taken with a Nikon microscope.

### Phagocytosis Assay

The phagocytosis assay was performed with procedures reported (Liu et al., 2018). In short, the photoreceptor outer segments (POSs) were collected from swine retina, labeled with CM-Dil (Invitrogen, CA, United States) following the instructions. Pigmented RPE cells differentiated from both patient and control iPSCs were cultured in RDM for 10 weeks after passage, and then treated with the CM-DiI labeled POS at 37°C or 4°C for 12 h. Afterward, RPE cells were washed with PBS thoroughly, fixed by 4% PFA for 5 min, and immunostained with ZO-1, a tight junction marker, to further determine the POS internalization. Z-stack images were taken with a Zeiss LSM 880 confocal microscope (Carl Zeiss Meditec, Inc.). For quantitative analysis, POS with a minimum diameter of 0.5 μm was counted using image J software, and five random fields of view (40×) were photographed per group. Three independent experiments were conducted.

### Enzyme-Linked Immunosorbent Assay

Monolayered RPE cells, grown on Matrigel-coated 24-well plates for 10 weeks, were used to evaluate VEGF secretion. After PBS washing, the cells were cultured in 500 μl DMEM-basic for 24 h, then the media were collected and centrifuged at 300 × *g* for 5 min. Total secreted VEGF in culture medium was assayed using a human VEGF Enzyme-Linked Immunosorbent Assay (ELISA) kit (QuantiCyto, China) following the manufacturer’s instructions. The RPE cells in each well after medium collection were digested into single cells, and then counted. The VEGF amounts secreted by 1 × 10^6^ RPE cells per well were used to compare between the patient and control groups.

### Statistical Analysis

All the results are presented as the mean ± *SD*. Comparisons between two groups were analyzed using a two-tailed Student’s *t*-test. *P* < 0.05 was considered statistically significant.

## Results

### Reprogram RPE65-Patient Urine Cells Into iPSCs

To non-invasively acquire somatic cells for reprogramming, we collected 100 ml middle stream of the micturition from one 9-year old RPE65-LCA patient, from which UCs were isolated and cultured as described previously (Xue et al., 2013; Supplementary Figure S1A). The obtained UCs were mixed with type 1 and 2 cells. The type 1 cells were rounded and grew closely in colonies while the type 2 were elongated and grew sparsely or surrounding type 1 cells (Supplementary Figure S1B). They had high proliferative capacity and could expand for more than 5 passages. As assessed by immunofluorescence staining, these cells expressed UC-specific proteins E-cadherin, CD44, and the intermediate filament keratin 7 (KRT7) (Supplementary Figure S1C). The morphological and molecular features of UCs from the RPE65-LCA patient were similar to those from healthy individuals reported previously (Zhou et al., 2011).

**FIGURE 1:**
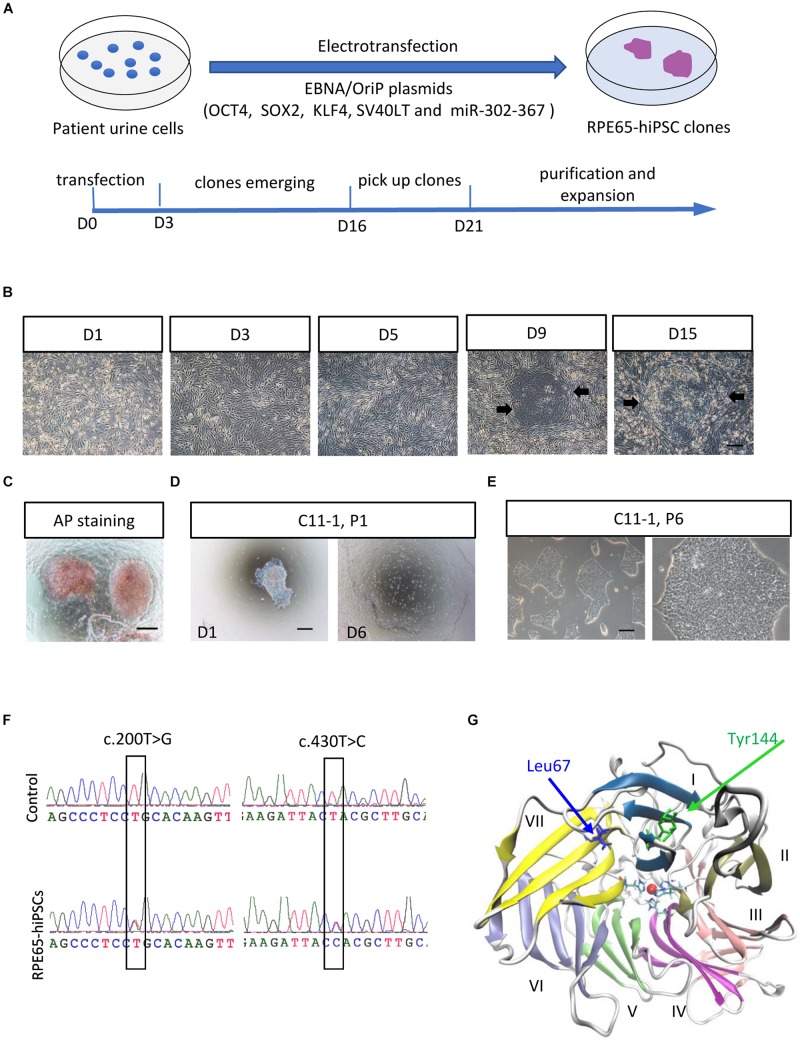
Generation and amplification of RPE65-hiPSCs. **(A)** Schematic overview of generating integration-free RPE65-hiPSCs from UCs. **(B)** Progressive formation of RPE65-hiPSCs colonies after UCs reprogrammed. The black arrows showed emerging clones. **(C)** The emerging clones were positive for AP staining. **(D)** A manually picked small clump from primary clone gradually formed typical clones on D6. **(E)** Passaged RPE65-hiPSCs clones with EDTA treatment. Scale bars, 250 μm. **(F)** Sanger sequencing confirmed hiPSCs from the LCA patient contained compound RPE65 gene mutations with c.200T>G and c.430T>C. **(G)** Structure of human RPE65 monomer viewed from the bottom face of the seven-bladed β-propeller. Blades are numbered I–VII. Absolutely conserved His180, His241, His313, and His527 residues are shown as sticks coordinating the natively bound iron ion. The two mutation sites Leu67 (L67) and Tyr144 (Y144) are shown as blue and green sticks, respectively.

To obtain non-integrating hiPSCs, we transfected OSTK factors (OCT4, SOX2, SV40T, and KLF4) along with miR-302-367 cluster expressing episomal plasmids into UCs through electroporation manner (Figure 1A). The transfected UCs cultured on Matrigel-coated dishes appeared a few of clonal cell clusters in 10 days, which became larger in size over time (Figure 1B and Supplementary Figure S3A). In 3 weeks after transfection, many flat and tightly packed colonies with clear boundary presented. AP staining showed that this type of colonies was positive (Figure 1C). To efficiently purify these good colonies, we manually picked small pitches from primary colonies one by one and plated them on Matrigel-coated 24-well plates, respectively, containing mTeSR1. With this method, the selected pitches grew and showed highly homogeneous and flat morphology with a sharp edge without obvious differentiation after only one or two round selection (Figure 1D). After purification, these cell colonies could be routinely passaged with EDTA solution every 4–6 days, and kept typical morphological features with a high nuclear–cytoplasmic ratio and large nucleoli (Figure 1E), similar with human embryonic stem cells (hESCs) and hiPSCs derived from healthy individuals. Herein, we refer to these cells as RPE65-hiPSCs. More than 20 clones were obtained from two wells of reprogrammed UCs cultured in a six-well plate and five patient-specific hiPSC lines with more than 10 passages were established.

Sanger sequencing confirmed the RPE65-hiPSCs contained two heterozygous mutations c.200T>G (p. L67R) and c.430T>C (p. Y144H) in *RPE65* gene, consistent with mutations detected in blood sample of this patient reported previously (Chen et al., 2013; Figure 1F). Structural analysis showed that the mutation sites L67 and Y144 were located in the blade VII and I of RPE65, respectively, which are critical to the closure of the core propeller fold (Figure 1G). Mutations (L67R and Y144H) had significant impacts on the local spatial volume, electrostatic potential, and/or hydrogen bonds network (Supplementary Figure S2). These structural effects may destabilize the protein, and thus result in improper “sealing” of the propeller structure or displace the critical iron-coordinating residues such as His527 and His180, reducing the enzyme activity.

### Characterization of RPE65-Patient-Specific hiPSCs

**FIGURE 2:**
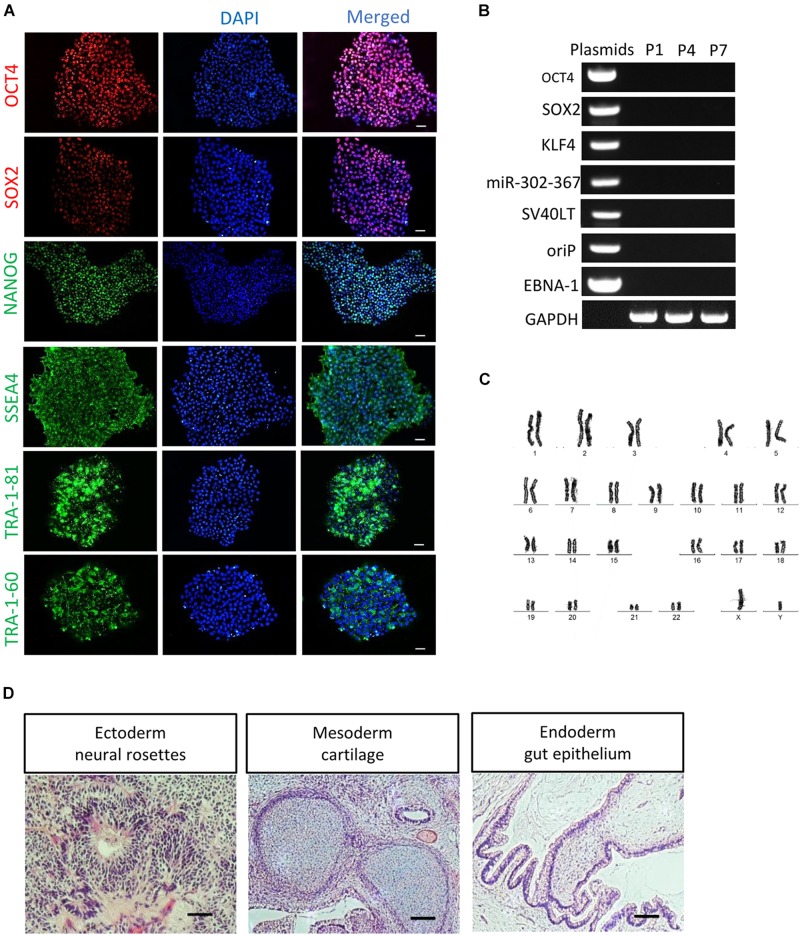
Characterization of non-integrated RPE65-patient-specific hiPSCs. **(A)** Immunofluorescence staining of pluripotency markers (OCT4, SOX2, NANOG, SSEA4, TRA-1-81, and TRA-1-60) for RPE65-hiPSCs (C11–1, P5). Scale bars, 50 μm. **(B)** Non-integrating analysis of episomal vectors in the RPE65-hiPSCs (C11–1, P1, P4, and P7) by RT-PCR. **(C)** G-band analysis showed that RPE65-hiPSCs (representative result of C11–1) had normal karyotype. **(D)** HE staining analysis of teratomas from RPE65-hiPSC in NOD-SCID mice. Scale bars, 100 μm.

After serial passage, the established cultures of RPE65-iPS cells were subjected to stringent assessment of characteristics of human pluripotent stem cells through different assays. Immunofluorescence staining showed these cells expressed human ESC/iPSC-specific protein markers OCT4, SOX2, NANOG, SSEA4, TRA-1-81, and TRA-1-60 (Figure 2A and Supplementary Figure S3C). Meanwhile, RT-PCR verified all five RPE65 hiPSCs lines (C11–1, C4, C10, C13, and C14) were positive for endogenous pluripotent genes *OCT4*, *SOX2*, and *NANOG* (Supplementary Figure S3B), but negative for exogenous reprogramming factors (OCT4, SOX2, SV40T, KLF4, and miR-302-367), and episomal plasmid DNA (oriP and EBNA-1), which were detected negatively in as early as passage one (P1) cells (Figure 2B and Supplementary Figure S3D). These results indicated RPE65-LCA patient-specific hiPSCs are free of episomal DNA integration, benefiting future translational study such as cell transplantation. RPE65-hiPSCs from two lines (C11–1, C4) at different passage number were chosen to analyze karyotype. They all showed normal karyotype by G-band staining (Figure 2C and Supplementary Figure S3E). To evaluate the pluripotency, we performed teratoma formation assays. The RPE65-hiPSCs were injected into NOD/SCID mice and followed up for 2 months. The patient-specific hiPSCs developed teratoma which contained neural rosettes, cartilage, and gut-like epithelium from three germ layers, respectively (Figure 2D). The above results demonstrated that RPE65-LCA patient-specific hiPSCs had pluripotency features similar to hESCs *in vitro* and *in vivo*.

### Generation of Patient-Specific Retinal Organoids From RPE65-hiPSCs

**FIGURE 3:**
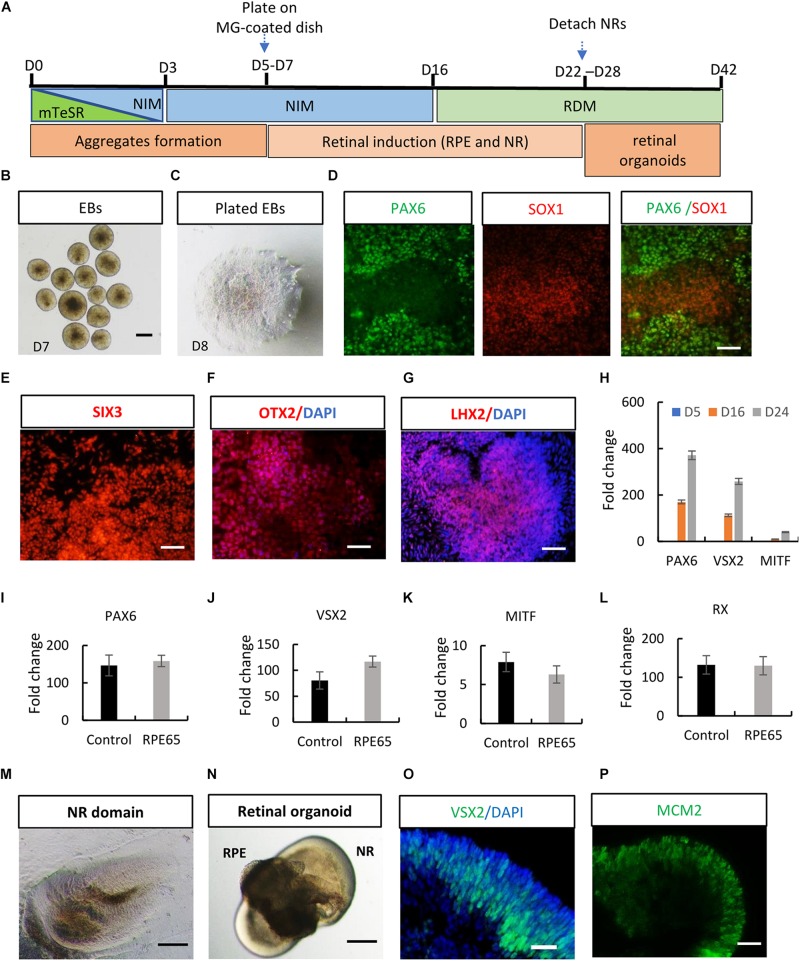
Acquisition of retinal organoids and RPE cells from RPE65-hiPSCs. **(A)** Schematic overview of retinal differentiation protocol. **(B)** Floating embryoid bodies (EBs). **(C)** EBs plated on Matrigel-coated dishes on D7. Scale bars, 200 μm. **(D)** The adherent EBs acquired an anterior neuroepithelial fate characterized by PAX6 and SOX1 expression. Scale bar, 50 μm. **(E–G)** SIX3, OTX2, and LHX2-positive retinal progenitor cells appeared around D12. Scale bars, 20 μm. **(H)** qRT-PCR showed progressive increase of expression level of retinal progenitor markers PAX6 and VSX2 and RPE marker MITF during retinogenesis of RPE65-hiPSCs. RPE65-hiPSCs derived EBs (D5) as reference. Mean ± *SD*, *n* = 3. **(I–L)** Comparison of mRNA expression level of retinal progenitor markers (PAX6, VSX2, and RX) and RPE marker (MITF) between control and RPE65 hiPSCs on D16 after retinal differentiation. Control hiPSCs derived EBs (D5) as reference. No significant difference was disclosed between them (*P* > 0.05), mean ± *SD*, *n* = 3. **(M)** Typical morphology of horseshoe-like NR domains. Scale bar, 200 μm. **(N)** Representative image of retinal organoids containing NR and RPE. Scale bar, 200 μm. **(O,P)** Immunofluorescence staining showed retinal progenitor cells expressing VSX2 and MCM2 occupied the whole neural retina on D35 after differentiation. Scale bars, 20 μm.

After stringent characterization of RPE65-hiPSCs, we asked whether they were able to differentiate into retinal organoids which were achieved with hiPSCs from healthy individuals (Zhong et al., 2014; Li et al., 2018). Using a stepwise retinal differentiation protocol reported (Li et al., 2018), the patient-specific hiPSCs recapitulated the major molecular and cellular features of retinal morphogenesis *in vivo*. Under suspension culture condition, dissociated RPE65-hiPSCs gradually formed EBs, which were then plated onto Matrigel-coated dishes for further induction (Figures 3A–C). Under these conditions, the RPE65-hiPSCs sequentially acquired PAX6^+^ and SOX1^+^ anterior neuroepithelial (AN) cell fate (Figure 3D) and Eye Field (EF) cell fate expressing EF transcription factors SIX3, OTX2, and LHX2 in 2 weeks after differentiation (Figures 3E–G). In addition, qRT-PCR showed that expression level of retinal progenitor markers (PAX6 and VSX2) and RPE cell markers (MITF) dramatically increased as differentiation progressed (Figure 3H). Compared to the healthy control hiPSCs, RPE65-hiPSCs presented similar mRNA expression level of retinal-specific genes *PAX6*, *VSX2*, *MITF*, and *RX* on D16 after induction (*n* = 3) (Figures 3I–L). Afterward, highly reflective, horseshoe shape like NR domains progressively formed, which were readily identified under inverted microscope (Figure 3M). Four weeks after differentiation, the domains were detached and cultured in suspension condition, under which they self-formed 3D retinal organoids with a major part of transparent NR ring attached with a small RPE ball on the other side, resembling an eye-cup (Figure 3N). The NR cells extensively expressed retinal progenitor markers VSX2 and MCM2 (Figures 3O,P). At least three RPE65-iPSCs lines (C4, C11-1, and C13) were successfully tested for their retinal differentiation ability. Collectively, these data indicated that RPE65-LCA patient-specific hiPSCs had capacity to form retinal organoids with the similar manner as hiPSCs derived from healthy individuals.

### Differentiation and Lamination of Patient-Specific Neural Retina From RPE65-hiPSCs

**FIGURE 4:**
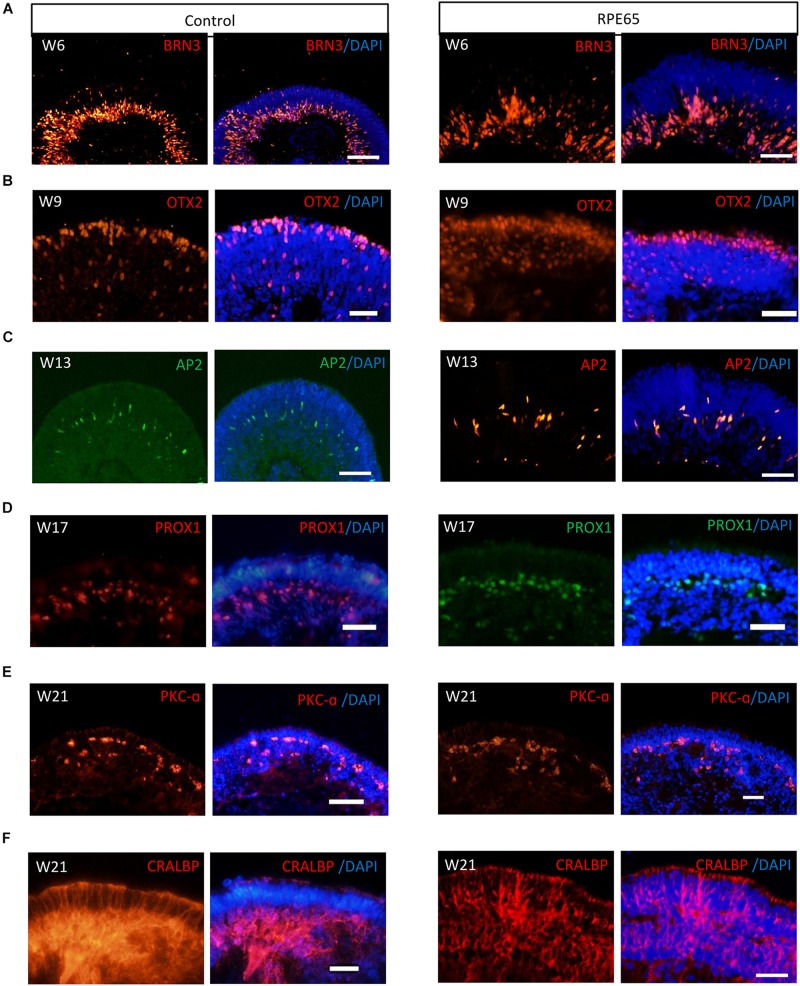
RPE65-hiPSCs generated layered retinal organoids with all retinal cell types. Comparable to healthy control, neural retina in RPE65-hiPSCs derived retinal organoids developed laminated architecture with all major retinal cell types located in the corresponding layer. **(A)** BRN3^+^ retinal ganglion cells located in the basal side, **(B)** OTX2^+^ photoreceptor cells lying in the apical side, **(C,D)** AP2^+^ amacrine cells and PROX1^+^ horizontal cells lying in intermediate layer, **(E)** PKC-a^+^ bipolar cells, and **(F)** CRALBP^+^ Müller glial cells. Scale bars, 20 μm.

Our pervious study showed that retinal progenitor cells (RPCs) differentiated from healthy urine-derived hiPSCs had ability to differentiate into all major retinal cell types in an ordered fashion that retinal ganglion cells are born first, followed by photoreceptor cells, amacrine cells, horizontal cells, and lastly by bipolar cells and Müller cells (Li et al., 2018). Here, we, for the first time, demonstrated that patient RPE65-hiPSCs could also recapitulate the spatiotemporal pattern of NR differentiation *in vivo*. RPE65-hiPSCs derived 3D retinal organoids comprised NR attached with more or less RPE. Retinal ganglion cells expressing BRN3 first appeared in W6 and located in the basal-most zone of the NRs (Figure 4A). Subsequently, the OTX2^+^ photoreceptor progenitor cells appeared and occupied the apical part of NRs in W9 (Figure 4B). At the mid-term stage (W12–17) appeared interneurons, including AP2^+^ amacrine cells and PROX1^+^ horizontal cells lying in the intermediate layer of the NRs (Figures 4C,D). Finally, the developing late-born neurons appeared in W18–21, such as PKC-a^+^ bipolar cells and CRALBP^+^ Müller glial cells (Figures 4E,F). These results demonstrated that patient RPE65-hiPSCs kept the capacity to generate laminated NR with the similar developmental order as those from the control hiPSCs. In addition, all of three RPE65-iPSCs lines (C4, C11-1, and C13) could generate well-layered retinal organoids.

### Photoreceptor Subtype Specification in Patient Retinal Organoids

**FIGURE 5:**
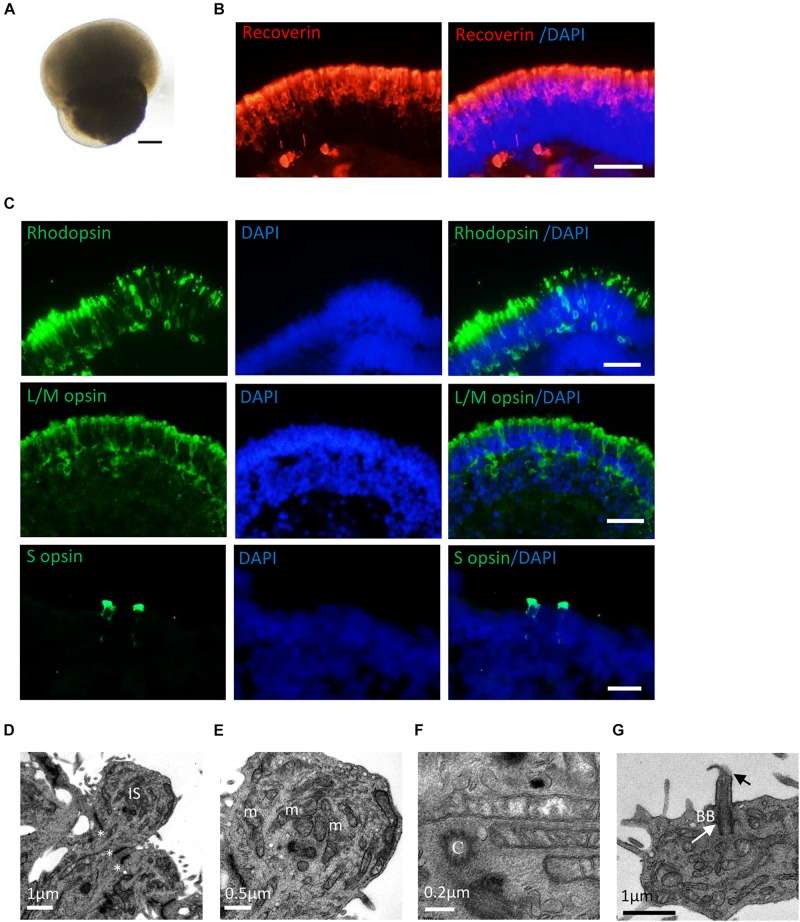
Photoreceptor subtype specification in patient retinal organoids. **(A)** Representative images of retinal organoids derived from RPE65-hiPSCs on D150. Scale bars, 200 μm. **(B)** Photoreceptor cells developed outer nuclear layer-like structure in patient retinal organoids, expressing photoreceptor cell pan marker Recoverin. Scale bar, 20 μm. **(C)** The patient photoreceptor cells had capacity to develop all subtypes including Rhodopsin^+^ rods, L/M opsin^+^ red/green cones and S opsin^+^ blue cones. Scale bars, 20 μm. **(D–G)** TEM revealed that RPE65-hiPSC-derived photoreceptors developed typical ultra-structures, outer limiting membrane (OLM, asterisks), inner segment (IS) with rich mitochondria (m), centriole (C), basal body (BB) (white arrow), and connecting cilia (black arrow). Scale bars, 1 μm **(D)**, 0.5 μm **(E)**, 0.2 μm **(F)**, and 1 μm **(G)**.

After long-term culture (>20 weeks), RPE65-hiPSCs derived 3D retinal organoids kept nice structure comprising a high-reflective NR tissue attached a small RPE patch (Figure 5A). Immunofluorescence showed that RPCs could differentiate into recoverin positive photoreceptor cells accumulated in the apical side and forming presumptive ONL (Figure 5B and Supplementary Figure S4). By W21, all subtypes of photoreceptors, including Rhodopsin^+^ rods, L/M opsin^+^ red/green cones, and S opsin^+^ blue cones were also acquired and self-organized with polarization of segments toward the apical side of the NRs (Figure 5C). In addition, TEM demonstrated that RPE65-hiPSCs derived photoreceptors developed typical ultrastructures, including outer limiting membrane, inner segment rich of mitochondria, basal body, connecting cilium, centriole, and rudimentary outer segment (Figures 5D–G). These results implied that RPE65-hiPSCs could generate all subtypes of photoreceptors in organoids, similar to those derived from the healthy hiPSCs (Li et al., 2018).

### Down-Regulation of RPE65 in Patient-Specific RPE Cells With Novel *RPE65* Mutations

**FIGURE 6:**
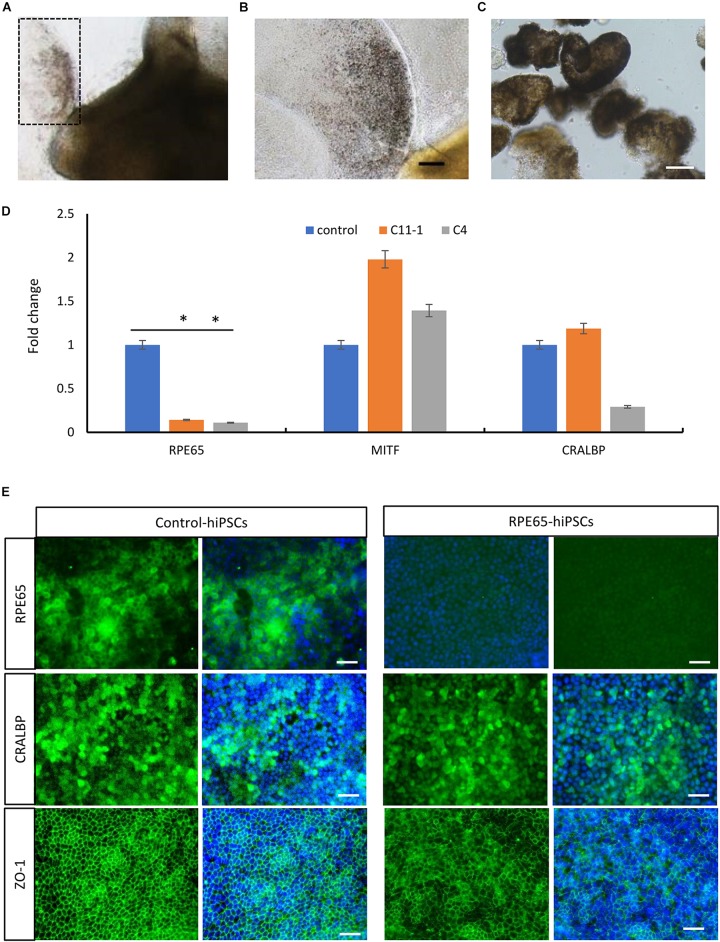
Decreased expression of RPE65 in patient RPE cells. **(A)** Pigmented RPE cells (dashed area) differentiated from RPE65-hiPSCs. **(B)** Higher magnification of the dashed area in **A**. Scale bar, 100 μm. **(C)** The picked RPE sheets on W8 after differentiation. Scale bar, 100 μm. **(D)** qPCR analysis showed that the expression level of RPE65 was significantly lower in patient RPE cells (C11–1 and C4) than in the control (UE022), while MITF and CRALBP in patient RPE cells were comparable to the control. ^∗^*P* < 0.05. Data presented as mean ± *SD*, *n* = 3. **(E)** Immunofluorescence staining revealed that RPE65 were negative in RPE65-hiPSCs derived RPE cells, but positive in control hiPSCs derived RPE cells, while both CRALBP and ZO-1 were strongly positive in patient and control RPE cells. Scale bars, 20 μm.

By using the same 3D retinal differentiation protocol as described above, RPE cells were simultaneously generated with NR from hiPSCs, presented pigment and cobblestone morphology in adherent culture as early as W4 after differentiation (Figures 6A,B). On W8, the pigmented RPE sheets derived from RPE65-hiPSCs and control were collected to test mRNA expression level of RPE65, MITF (the RPE cell transcription factor), and CRALBP (cellular retinaldehyde-binding protein) (Figure 6C). qRT-PCR showed that RPE65 expression level was significantly lower (approximately eightfold) in RPE cells from RPE65-hiPSCs than those from control hiPSCs (*P* < 0.05). However, MITF and CRALBP expression levels had no statistically significant difference between RPE65-hiPSCs and control hiPSCs derived RPE (Figure 6D). Moreover, immunofluorescence staining revealed that the RPE65 protein expression was hardly detected in RPE65-hiPSCs derived RPE cells at W8 after differentiation, but in control RPE cells. While CRALBP and ZO-1, a tight junction protein, were positive in RPE cells from both control and RPE65-hiPSCs (Figure 6E). These results indicated the compound RPE65 mutations L67R and Y144H down-regulated RPE65 mRNA and protein expression in patient-specific RPE cells.

### Expansion Capacity of Patient-Specific RPE Cells

In addition, whether *RPE65* gene mutations impair the expansion capacity of patient RPE cells were evaluated. RPE sheets from control and RPE65-hiPSCs with pigmentation and cobblestone-like morphology were selected, and digested into single cells on W8 after differentiation, and then seeded on Matrigel-coated dishes for expansion (Supplementary Figures S5A,B). In RCM containing serum, RPE65-hiPSCs derived pigmented RPE cells proliferated, depigmented, and reached confluency in 1 week when cell plating density was 2–5 × 10^4^/cm^2^. The cells could passage every week and expanded more than three passages, yielding large populations which could be frozen and revived for future applications. When switched to RDM without serum, the expanded RPE cells regained pigmentation with typical cobblestone-like morphology, signs of mature RPE cells (Supplementary Figure S5C). Immunofluorescence staining showed that these RPE cells expressed RPE-specific markers PAX6, OTX2, MITF, and the tight junction marker ZO-1, comparable to the RPE cells derived from control hiPSCs (Supplementary Figure S5D).

### Evaluation of Cell Function of Patient RPE

Next we asked whether the novel *RPE65* mutations L67R and Y144H impacted on RPE cell function. POS phagocytosis and VEGF secretion were evaluated in RPE cells derived from both control and RPE65-hiPSCs. Compared to those cultured on 4°C condition (as a negative control, normal physiological function reduced at this temperature), all RPE cells on 37°C could phagocytose POS actively. In addition, the POS number phagocytosed by the control and patient RPE cells were comparable (142.8 ± 9.4/view and 175.7 ± 7.6/view, *n* = 3, respectively), implying the RPE65 mutations did not change phagocytosis capacity of patient RPE cells (Supplementary Figures S6A–C). By ELISA analysis, the VEGF amounts secreted by patient RPE cells (547.7 ± 17.3 pg/10^6^ cells, *n* = 3) were equivalent to the control RPE cells (585.2 ± 23.0 pg/10^6^ cells, *n* = 3) (Supplementary Figure S6D). Therefore, the above data indicated that the compound RPE65 mutations L67R and Y144H had no effect on the two important biological function of patient RPE cells. However, further studies will be needed to elucidate whether the novel mutations reduce the RPE65 isomerase activity, critical for the regeneration of the visual pigment and normal vision.

## Discussion

In this study, we generated non-integrating hiPSCs from a presumptive LCA patient carrying novel *RPE65* mutations c.200T>G (p. L67R) and c.430T>C (p.Y144H). These patient-specific hiPSCs had typical features of healthy hiPSCs, such as clonal growth, expression of pluripotent markers, and multipotential differentiation. Moreover, RPE65-hiPSCs could differentiate into retinal organoids containing photoreceptor and RPE cells, two disease target cells. The time course of retinal differentiation and cellular and structural features of retinal organoids from RPE65-hiPSCs was similar to those from control hiPSCs. In addition, our findings disclosed that the patient RPE cells had normal biological functions, POS phagocytosis, and VEGF secretion, but lower expression of RPE65 mRNA and protein. The latter might reduce the RPE65 enzyme activity, leading to the vision loss.

The retinoid isomerase, RPE65, exclusively expressed in RPE cells, is critical for visual cycle responsible for sustaining vision. Over 130 genetic variants locating in coding or non-coding regions of this gene have been reported in LCA patients with diverse clinical phenotypes (Astuti et al., 2016). In the past decades, significant progress has been achieved in understanding normal RPE65 action and disease pathogenic mechanisms as well as developing *RPE65* gene augment therapy (Redmond et al., 1998; Jin et al., 2005; Russell et al., 2017). So far, different animal models with *RPE65* knockout, point mutations, or naturally inactivating mutations of *RPE65* (mice and dog) have been established or identified, and made huge contributions to the above achievements (Li et al., 2014; Shin et al., 2017). However, species issues in model fidelity and response to therapeutic treatments existed. Bainbridge et al. (2015) reported that gene therapy with rAAV2/2 *RPE65* vector resulted in only modest and temporary improvement in LCA patients compared with results obtained from the dog model. They postulated that there was a species difference in the amount of RPE65 required to drive the visual cycle (Bainbridge et al., 2015). Hence, there is a need to develop a human *in vitro* model of LCA caused by RPE65 for basic and translational study. With hiPSC technology, Tucker et al. (2015) created patient hiPSCs lines containing exonic leucine–toproline mutation (L408P) and intron 3 (IVS3-11) mutation in *RPE65* gene, induced them into patient-specific RPE cells from which the IVS3-11 variation was disclosed causing mis-splicing via transcriptional analysis. However, the direct impact of the above mutations on RPE65 expression, enzyme activity, and RPE cell function remains unclear. Here, we produced patient-specific hiPSCs which differentiated into retinal cells including RPE cells from a putative LCA patient containing two novel *RPE65* mutations L67R and Y144H. The pathogenicity of these two compound mutations in RPE65 gene has not determined yet. Bioinformatic analysis predicted the L67R mutation was damaging or probably damaging, while the Y144H mutation tolerated or probably damaging (Chen et al., 2013). In cellular level, our study, for the first time, revealed that these mutations decreased the expression of RPE65 mRNA and protein in patient RPE cells, which are also in agreement with the structure analysis of the mutants (Figure 1G and Supplementary Figure S2). However, further studies will be necessary to confirm the impact of these mutations on the isomerase activity.

Advance in retinal organoids induction techniques with hiPSCs provides a powerful research platform for dissecting mechanisms of retinal development and disease *in vitro*. These multi-layered retinal organoids contained nearly all major retinal cell types, especially photoreceptor and RPE cells, which are the most common, initially affected cells in retina of LCA patient. So far, retinal organoids derived from patient-specific iPSC were reported to model, at some degree, phenotypes of retinal dystrophy, such as *CEP290* and *NR2E3* gene mutation-related LCA (Parfitt et al., 2016; Deng et al., 2018). In this study, we firstly acquired RPE65-LCA patient-specific retinal organoids containing neural retinal and RPE in different developmental stages. In patient retinal organoids, all subtypes of photoreceptors including Rhodopsin^+^ rods, L/M opsin^+^, and S opsin^+^ cones developed and highly organized since W20 after differentiation. In addition, the protein expression pattern of these opsins in the organoids showed the similar spatial pattern as in the human fetal retina, with opsins first detected in the rudimentary OS, then in the entire cell membrane, and finally restricted to the elongating OS (Hendrickson et al., 2008). Especially, the typical ultrastructure of patient photoreceptors such as outer limiting membrane, inner segment, and rudimentary outer segments, a functional structure, also appeared. The success of acquiring rods and cones as well as RPE cells would provide a patient-specific cell model to dissect the pathogenic mechanisms and phenotype of the disease caused by these two mutations L67R and Y144H as well as other factors, personal factors in particular. However, a previous study showed that evident structural changes including outer segment discs were not seen in 7-week-old RPE65^–/–^ mice, but in 15-week-old RPE65^–^/^–^ mice (Redmond et al., 1998), implying the phenotype of photoreceptors presented quite late. Therefore, challenges with retinal organoids still exist in terms of modeling of photoreceptor phenotypes such as structural changes and cell loss caused by RPE65 mutations. Optimizing the retinal organoids culture system including promoting the photoreceptor maturation and long-term survival and establishing the direct contact of RPE and photoreceptors will be in demand for disease modeling of LCA.

Most of LCA patients inevitably suffer from retinal degeneration with thinning retina, which need tissue or cell replacement therapy. Although gene-based therapy could improve vision of RPE65-LCA patients, it failed to prevent degeneration process of this disease. Patient-derived retinal organoids or RPE cells can be utilized for regenerative medicine in combination with genome-editing technology (Deng et al., 2018; Zheng et al., 2018). Genome editing tools, the clustered regularly interspaced short palindromic repeats (CRISPR)/Cas9 system, zinc finger nucleases (ZFNs), and transcription activator-like effector nucleases (TALENs), are capable to correct mutations that lead to genetic diseases (Kime et al., 2016). With this technology, Soldner et al. (2011) created isogenic pairs of patient-derived iPSCs. Both RPE65-hiPSCs derived RPE cells and photoreceptor cells after mutation correction could provide unlimited seed cells for cell therapy alone or combined with gene therapy, especially in personalized therapy.

## Conclusion

In conclusion, we obtained patient-specific, integration-free RPE65-hiPSCs, and their derivatives of retinal organoids with photoreceptors and RPE containing RPE65 mutations L67R and Y144H. The patient-specific organoids with RPE and photoreceptor cells may serve as new biomaterials or cell disease models for precision medicine including personalized pathogenic mechanisms, drug screening, gene therapy evaluation, or cell replacement therapy. Further studies will be needed to evaluate the degree to which the patient-specific retinal organoids can mimic the disease progress, phenotypes, and molecular changes.

## Data Availability

The raw data supporting the conclusions of this manuscript will be made available by the authors, without undue reservation, to any qualified researcher.

## Ethics Statement

This study was approved by the ethics committee of the Zhongshan Ophthalmic Center of Sun Yat-sen University and was conducted in accordance with the Declaration of Helsinki. The patient agreed to take part in this experiment and signed informed consent.

## Author Contributions

GL and GG: collection and assembly of data, data analysis and interpretation, and manuscript writing. PW and QZ: provision of study material or patients. XS, PX, BX, TZ, and GP: collection and assembly of data. FP and JG: data analysis and interpretation. XZ: conception and design, data analysis and interpretation, manuscript writing, final approval of the manuscript, and financial and administrative support.

## Conflict of Interest Statement

The authors declare that the research was conducted in the absence of any commercial or financial relationships that could be construed as a potential conflict of interest.

## Footnotes

1 www.uniprot.org

## Supplementary Material

FIGURE S1 Collection and culture of urine cells from a RPE65-LCA patient. **(A)** Schematic diagram of urine sample collection and cell culture. **(B)** Representative phase contrast photographs of urine epithelium cells (UCs) at passage (P) 1. Arrow, type 1 cells; arrowhead, type 2 cells. Scale bars, 100 μm. **(C)** UCs expressed epithelium markers E-cadherin, CD44, and KRT7 by immunofluorescence staining. Scale bars, 20 μm.

FIGURE S2 Structural analysis of the *RPE65* mutations. **(A)** Hydrogen bonds analysis for the L67R mutation. Hydrogen bonds were shown as purple dash lines. There was no change in the local hydrogen bonds network when the mutation occurred. **(B,C)** The local electrostatic potentials within 3 Å of the 67th residues. Potentials less than −10 kT/e were colored in red, and those greater than +10 kT/e were depicted in blue. The positive charges around Arg67 were significantly higher than those of Leu67. **(D)** Hydrogen bonds analysis for the Y144H mutation. Y144H mutation lead to the loss of the hydrogen bond between Tyr144 and Asp 142. **(E,F)** The local electrostatic potentials within 3 Å of the 144th residues. His144 brought more positive charge than Tyr144.

FIGURE S3 Pluripotency and free-integration of RPE65-hiPSCs demonstrated by other clones. **(A)** A primary clone (C4) with clear boundary on D20 after reprogramming. Scale bar, 200 μm. **(B)** RT-PCR showed pluripotency gene expression (OCT4, SOX2, NANOG) of RPE65-hiPSCs from five different clones. **(C)** Immunofluorescence staining of pluripotency markers (OCT4, SOX2, NANOG, SSEA4, TRA-1-81, and TRA-1-60) for RPE65-hiPSCs (C4, P6). Scale bars, 50 μm. **(D)** RT-PCR showed negative expression of exogenous episomal plasmid DNA in five RPE65-hiPSCs lines tested [C11–1 (P7), C4 (P8), C10 (P8), C13 (P9), C14 (P8)]. **(E)** G-band analysis showed RPE65-hiPSCs (C4) had normal karyotype.

FIGURE S4 Negative controls of immunofluorescence staining (IFS) in Figure 5. To exclude the false positive caused by the non-specific binding of second antibodies, three types of second antibodies **(A–C)** used in Figure 5 were tested with PBS instead of the first antibodies, Recoverin raised from rabbit **(A)**, Rhodopsin from mouse **(B)**, and S opsin from rabbit **(C)** as the negative controls. IFS were performed parallelly on serial sections of retinal organoids older than W20. All images were taken under the same exposure conditions with an LSM 510 confocal microscope (Zeiss). The detailed information of all antibodies can be found in Table 1. Scale bars, 20 μm.

FIGURE S5 Propagation of RPE65-hiPSCs derived RPE cells. **(A)** Cobblestone-like RPE cells derived from RPE65-hiPSCs contained pigmentation 40 days after differentiation. Scale bar, 200 μm. **(B)** Passaged RPE cells on D2. Scale bar, 200 μm. **(C)** Passaged RPE cells presented cobblestone morphology and regained pigmentation 4 weeks after passage. Scale bar, 50 μm. **(D)** Immunostaining showed that the typical RPE markers PAX6, OTX2, MITF, and ZO-1 were positive in passaged RPE cells derived from both control and RPE65 hiPSCs. Scale bars, 20 μm.

FIGURE S6 Functional evaluation of patient RPE cells. **(A,B)** Z-stack confocal images showing the phagocytosed CM-Dil labeled POS (red) by RPE cells derived from control **(A)** and patient **(B)** hiPSCs. During 12 h POS incubation, cells cultured in 4°C were used as negative control. **(C)** The POS phagocytosis capacity of RPE65-hiPSCs derived RPE cells was comparable to the control. Mean ± *SD*, *n* = 3. **(D)** The total VEGF secretion of both control- and patient-derived RPE cells cultured for 24 h was equivalent. Mean ± *SD*, *n* = 3.
