# Supplementary figures and images for: Generation and Characterization of Induced Pluripotent Stem Cells and Retinal Organoids From a Leber’s Congenital Amaurosis Patient With Novel RPE65 Mutations
Source: Front Mol Neurosci. 2019 Sep 11;12:212. doi: 10.3389/fnmol.2019.00212 (PMC6749091; doi:10.3389/fnmol.2019.00212)

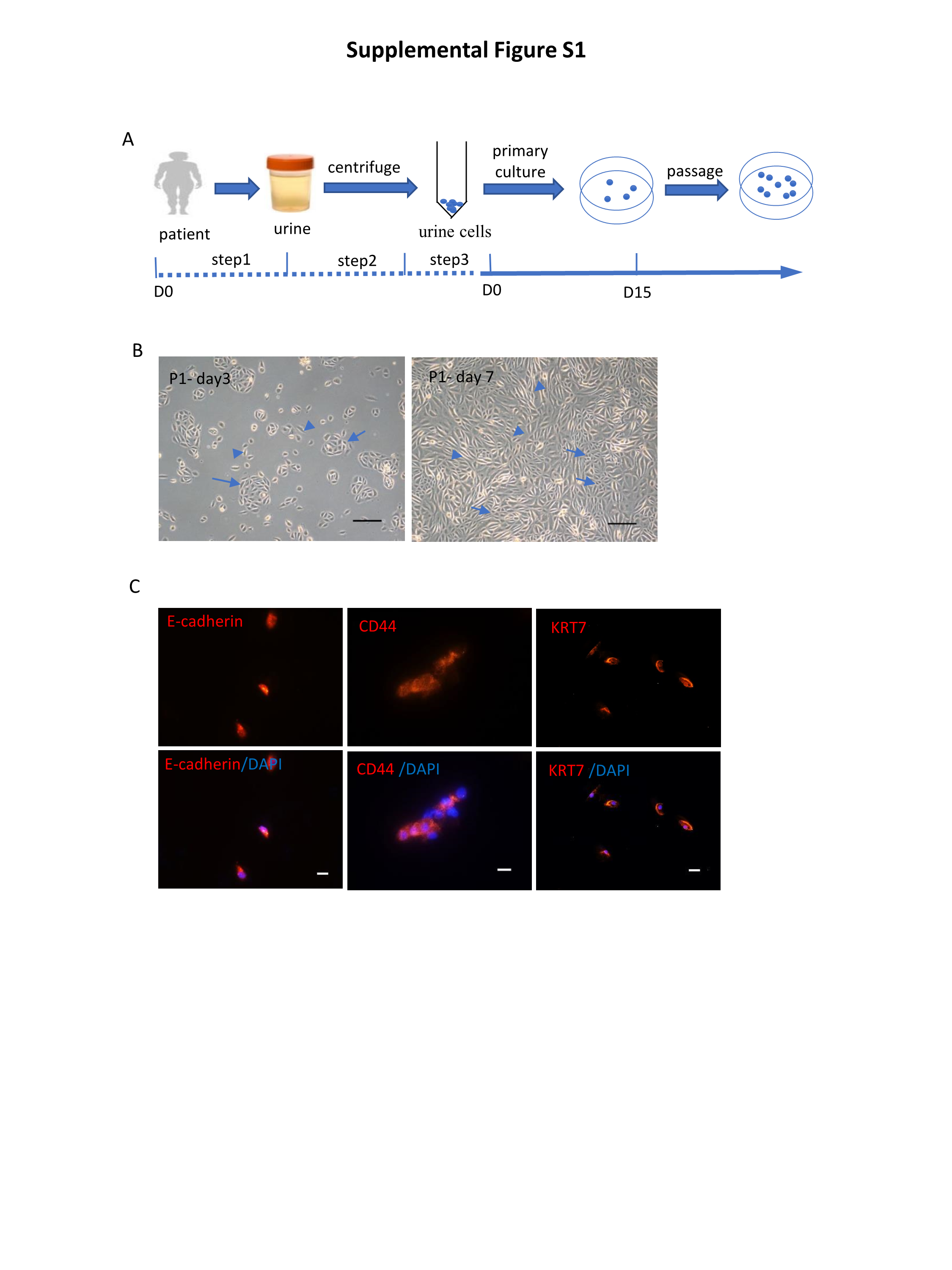

Supplement: FIGURE S1 — Collection and culture of urine cells from a RPE65-LCA patient. (A) Schematic diagram of urine sample collection and cell culture. (B) Representative phase contrast photographs of urine epithelium cells (UCs) at passage (P) 1. Arrow, type 1 cells; arrowhead, type 2 cells. Scale bars, 100 μm. (C) UCs expressed epithelium markers E-cadherin, CD44, and KRT7 by immunofluorescence staining. Scale bars, 20 μm. [file Image_1.TIFF]

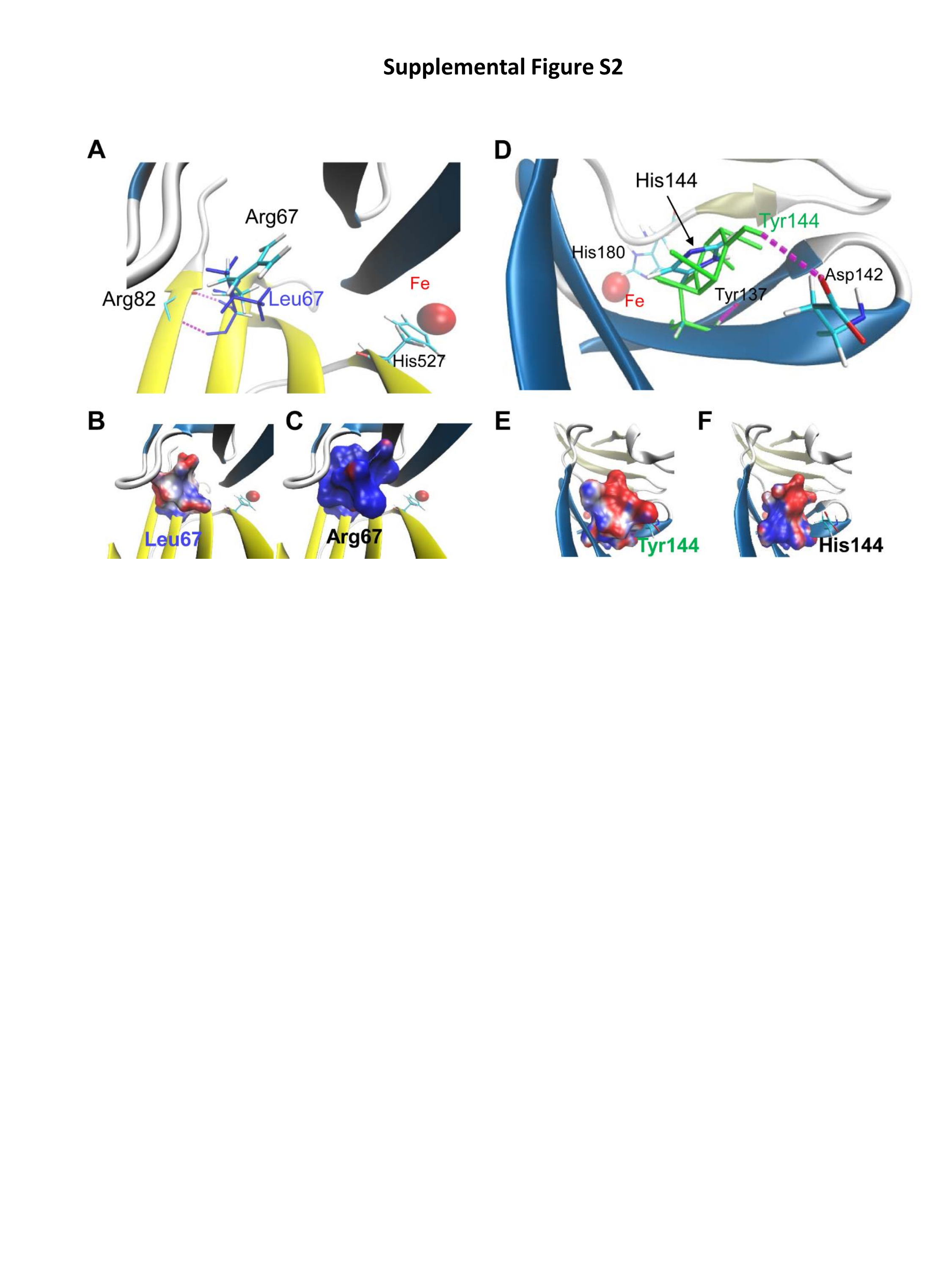

Supplement: FIGURE S2 — Structural analysis of the RPE65 mutations. (A) Hydrogen bonds analysis for the L67R mutation. Hydrogen bonds were shown as purple dash lines. There was no change in the local hydrogen bonds network when the mutation occurred. (B,C) The local electrostatic potentials within 3 Å of the 67th residues. Potentials less than −10 kT/e were colored in red, and those greater than +10 kT/e were depicted in blue. The positive charges around Arg67 were significantly higher than those of Leu67. (D) Hydrogen bonds analysis for the Y144H mutation. Y144H mutation lead to the loss of the hydrogen bond between Tyr144 and Asp 142. (E,F) The local electrostatic potentials within 3 Å of the 144th residues. His144 brought more positive charge than Tyr144. [file Image_2.TIFF]

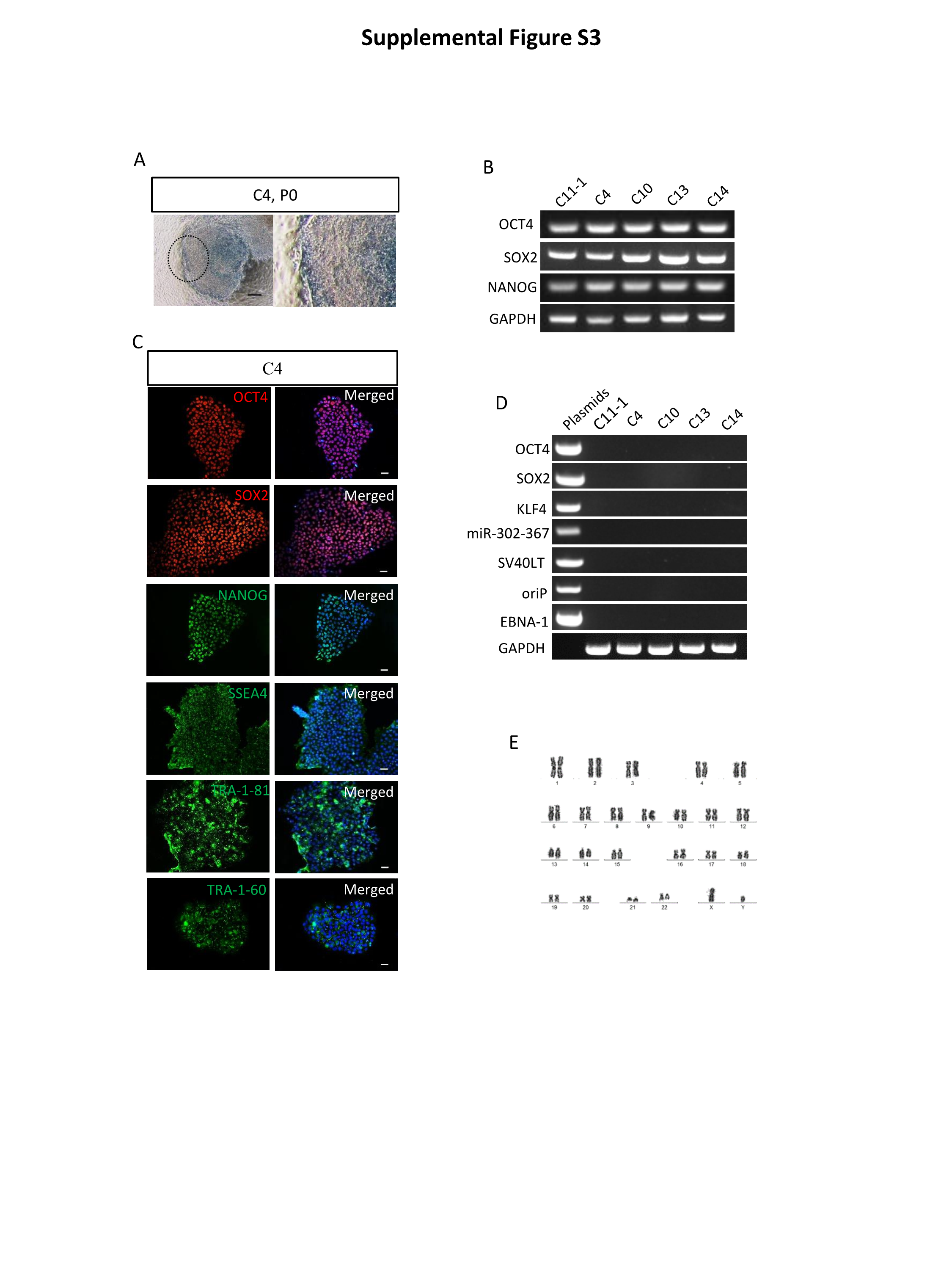

Supplement: FIGURE S3 — Pluripotency and free-integration of RPE65-hiPSCs demonstrated by other clones. (A) A primary clone (C4) with clear boundary on D20 after reprogramming. Scale bar, 200 μm. (B) RT-PCR showed pluripotency gene expression (OCT4, SOX2, NANOG) of RPE65-hiPSCs from five different clones. (C) Immunofluorescence staining of pluripotency markers (OCT4, SOX2, NANOG, SSEA4, TRA-1-81, and TRA-1-60) for RPE65-hiPSCs (C4, P6). Scale bars, 50 μm. (D) RT-PCR showed negative expression of exogenous episomal plasmid DNA in five RPE65-hiPSCs lines tested [C11–1 (P7), C4 (P8), C10 (P8), C13 (P9), C14 (P8)]. (E) G-band analysis showed RPE65-hiPSCs (C4) had normal karyotype. [file Image_3.TIFF]

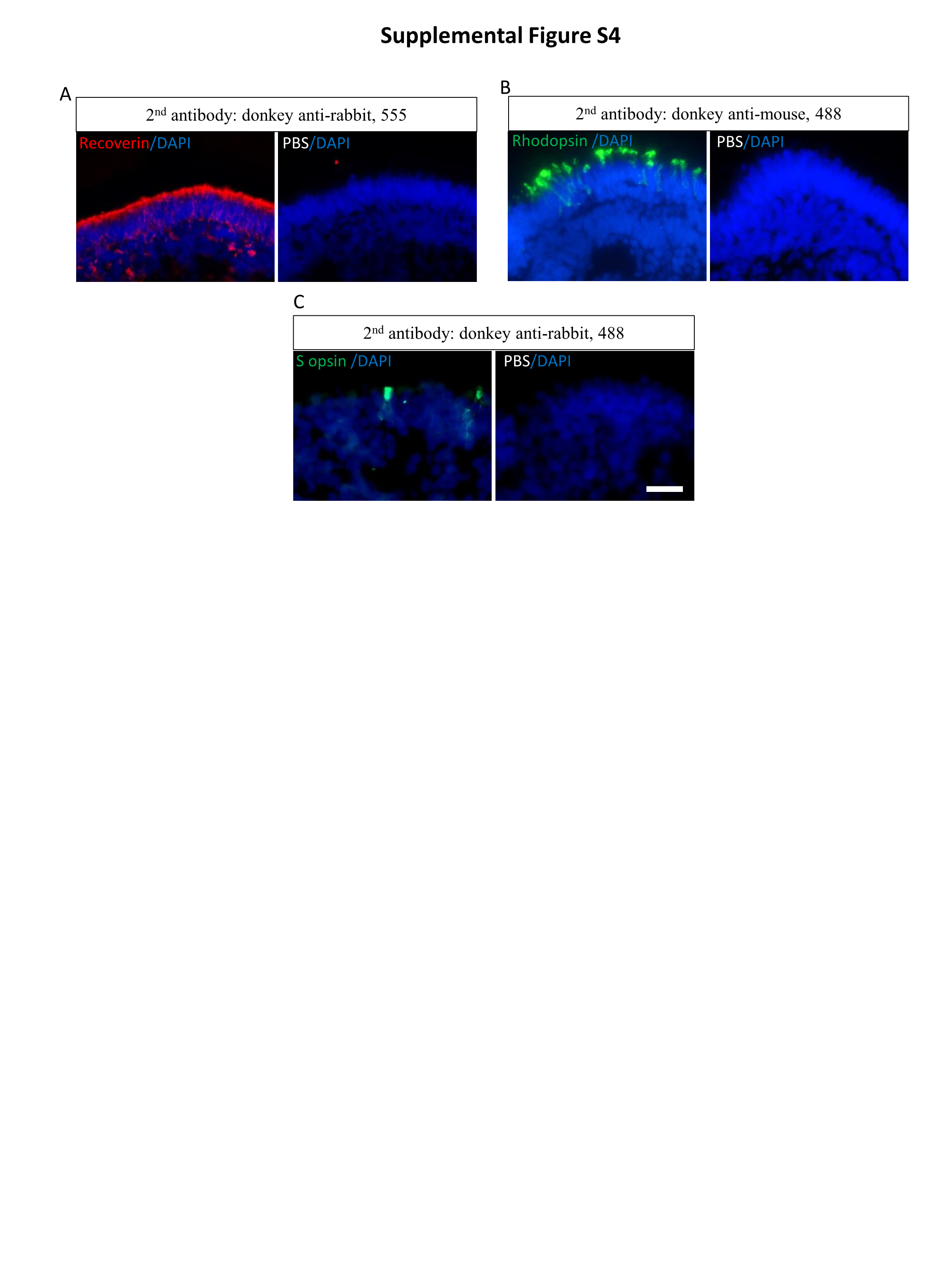

Supplement: FIGURE S4 — Negative controls of immunofluorescence staining (IFS) in Figure 5. To exclude the false positive caused by the non-specific binding of second antibodies, three types of second antibodies (A–C) used in Figure 5 were tested with PBS instead of the first antibodies, Recoverin raised from rabbit (A), Rhodopsin from mouse (B), and S opsin from rabbit (C) as the negative controls. IFS were performed parallelly on serial sections of retinal organoids older than W20. All images were taken under the same exposure conditions with an LSM 510 confocal microscope (Zeiss). The detailed information of all antibodies can be found in Table 1. Scale bars, 20 μm. [file Image_4.TIF]

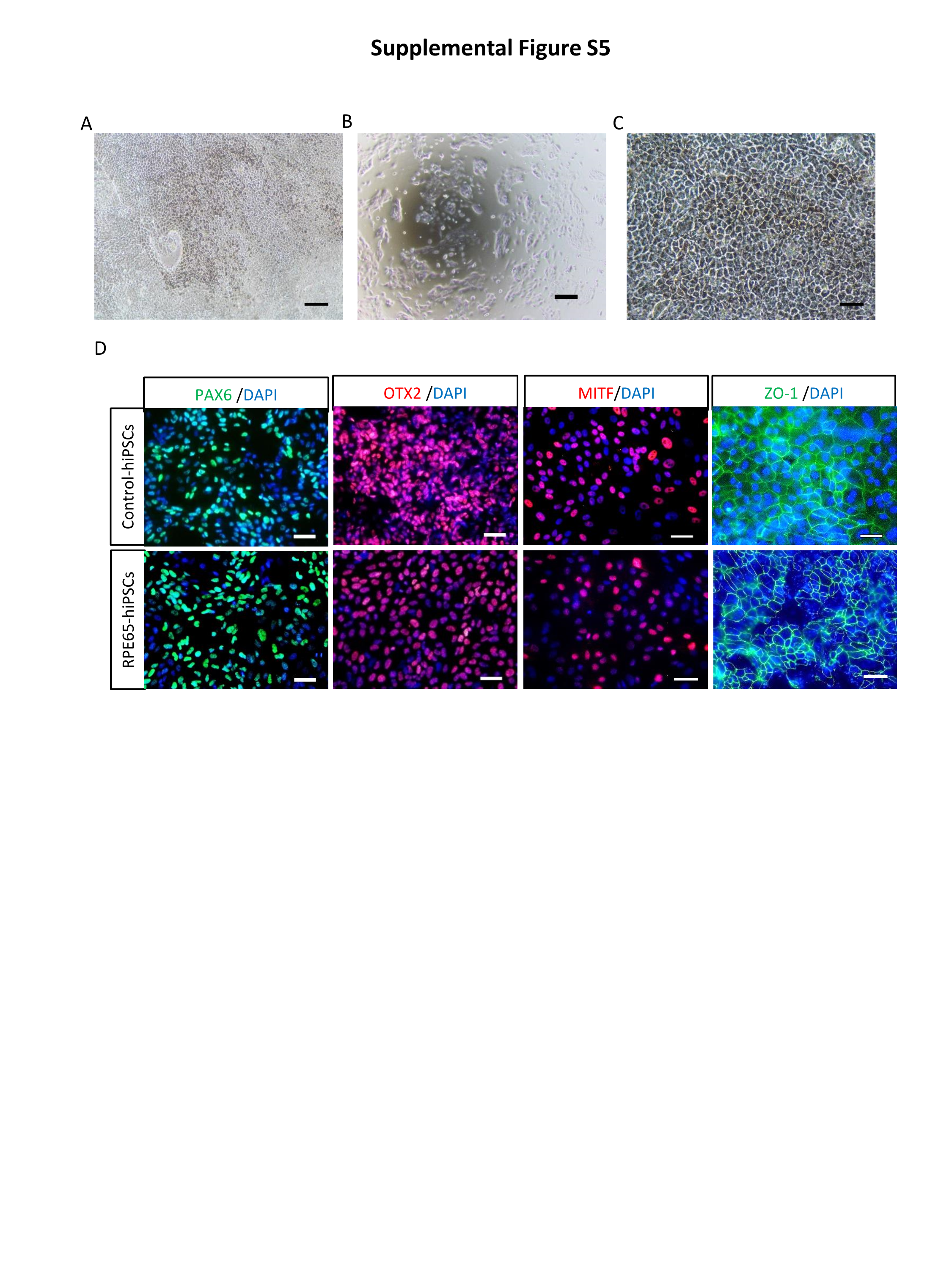

Supplement: FIGURE S5 — Propagation of RPE65-hiPSCs derived RPE cells. (A) Cobblestone-like RPE cells derived from RPE65-hiPSCs contained pigmentation 40 days after differentiation. Scale bar, 200 μm. (B) Passaged RPE cells on D2. Scale bar, 200 μm. (C) Passaged RPE cells presented cobblestone morphology and regained pigmentation 4 weeks after passage. Scale bar, 50 μm. (D) Immunostaining showed that the typical RPE markers PAX6, OTX2, MITF, and ZO-1 were positive in passaged RPE cells derived from both control and RPE65 hiPSCs. Scale bars, 20 μm. [file Image_5.TIF]

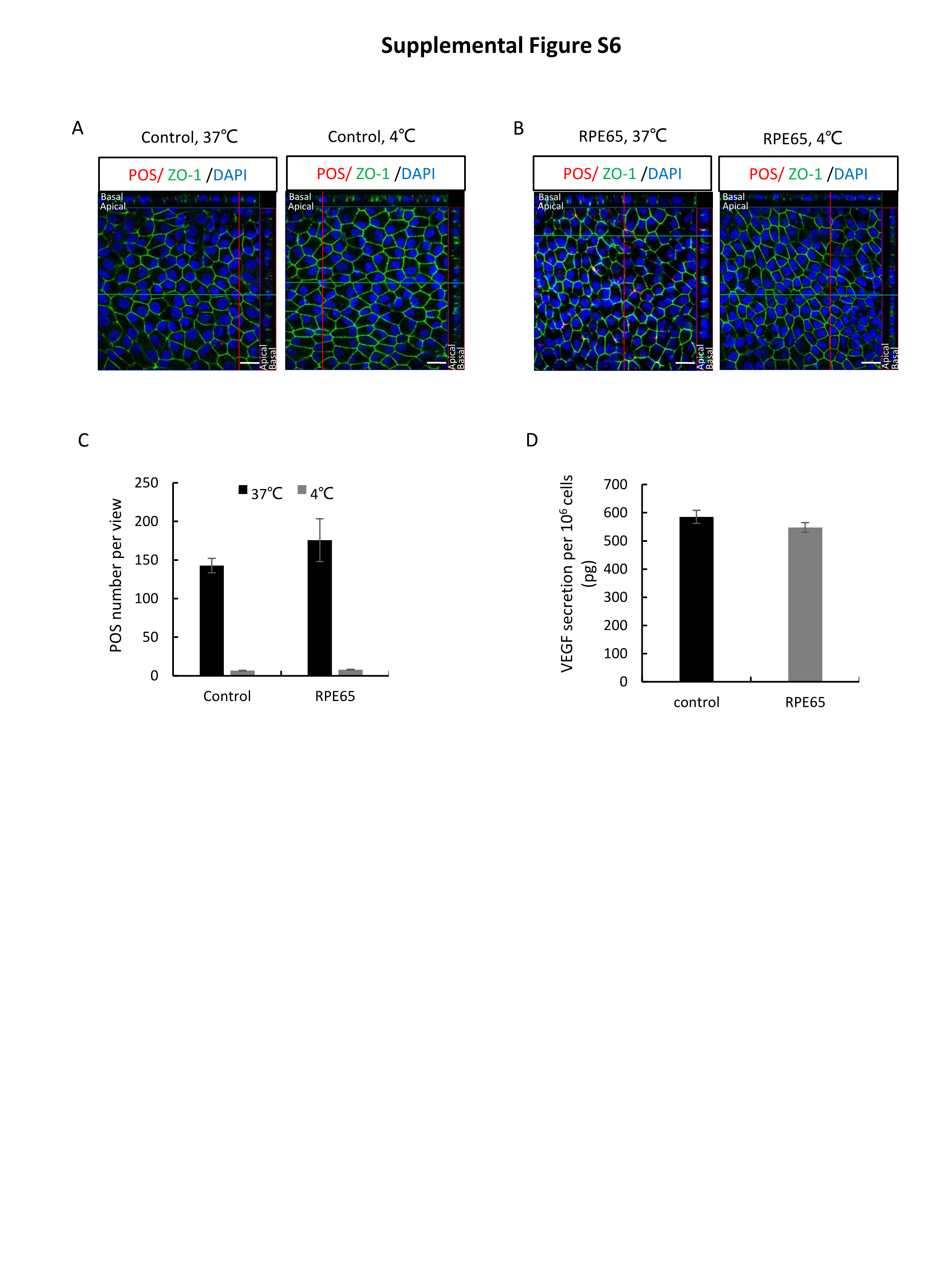

Supplement: FIGURE S6 — Functional evaluation of patient RPE cells. (A,B) Z-stack confocal images showing the phagocytosed CM-Dil labeled POS (red) by RPE cells derived from control (A) and patient (B) hiPSCs. During 12 h POS incubation, cells cultured in 4°C were used as negative control. (C) The POS phagocytosis capacity of RPE65-hiPSCs derived RPE cells was comparable to the control. Mean ± SD, n = 3. (D) The total VEGF secretion of both control- and patient-derived RPE cells cultured for 24 h was equivalent. Mean ± SD, n = 3. [file Image_6.TIF]
